# Multimodality Imaging Assessment of Rosai-Dorfman Disease: A Case Report

**DOI:** 10.1055/a-2592-1053

**Published:** 2025-09-17

**Authors:** Wenyue Cao, Cui Chen, Lan Ma, Xiaoyi Xie, Shulian Gu, Lili Du, Lan He, Yi Yu

**Affiliations:** 1 Department of Ultrasound 71141 Shanghai Jiao Tong University School of Medicine Shanghai Chest HospitalShanghaiChina; 2 Department of Ultrasound Medicine 71141 Shanghai Jiao Tong University School of Medicine Shanghai Chest HospitalShanghaiChina

A 34-year-old male presented with recurrent cough for half a year. He denied expectoration, chest tightness, dyspnea, palpitation, and hemoptysis. He had a smoking history and had quit smoking for 5 months. He had no family history of autoimmune disease or hepatitis. Physical examination showed abnormal breath sounds in the left chest. The complete blood count, C-reactive protein, blood levels of electrolytes, and immunoglobulin levels as well as the test results for liver and kidney function were normal.

**Fig. 1:**
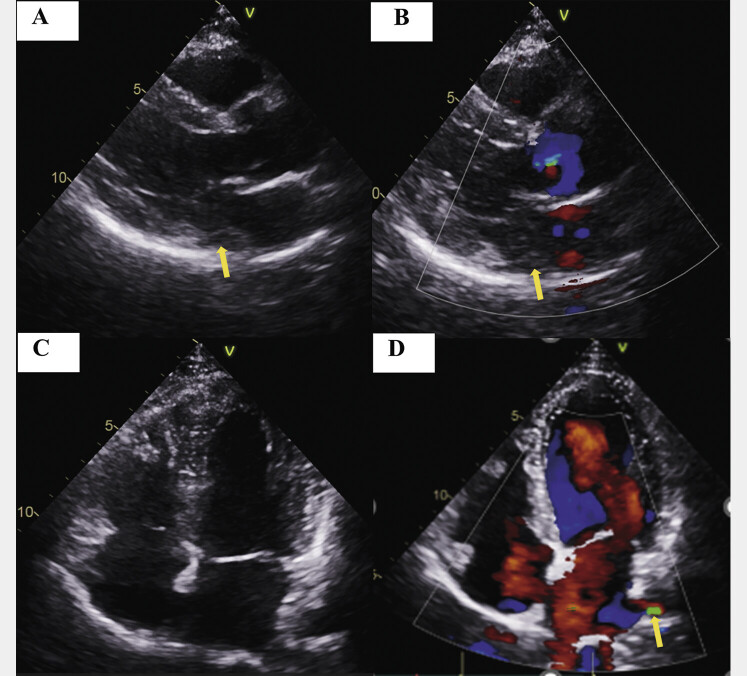
Transthoracic echocardiogram showed an occupying lesion in the pulmonary vein which extended into the left atrium. **A–B** From the parasternal long-axis view, the mass in the left atrium was detected (yellow arrow). **C** From the apical four-chamber view, the occupying lesions were not found in the pulmonary vein and left atrium. **D** Color Doppler flow imaging (CDFI) showed a colorful jet flow from the left inferior pulmonary vein (yellow arrow).

**Fig. 2:**
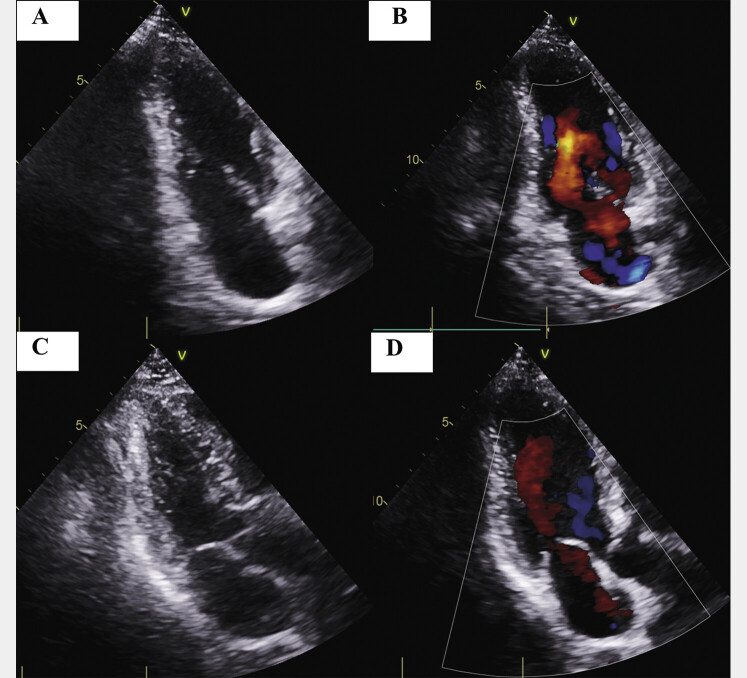
Transthoracic echocardiogram did not detect any occupying lesion from the apical two-chamber view ( **A, B** ) and three-chamber view ( **C, D** ).

On admission, initial 12-lead electrocardiogram demonstrated sinus bradycardia and the heart rate was 49 beats per minute. Contrast-enhanced computed tomography (CT) showed a 3.2cm round lesion enhancement in the lower lobe of the left lung close to the hilum involving vessels and the pleura. Hence, CT indicated the possibility of a sarcomatous transformation. Subsequently, transthoracic echocardiography (TTE) was performed. The left ventricle ejection fraction measured by Simpson’s method was 63%. The examiner was confronted with uncertain TTE results in the patient and missed the diagnosis in the beginning. While the images were reviewed by an echocardiography expert, an occupying lesion was discovered in the LA from the parasternal long-axis view ( Fig. 1 A–B), but it was not detected on the other views ( Fig. 1 C–D, Fig. 2 ). Furthermore, CDFI showed a colorful jet signal flow from the LIPV on the apical four-chamber view ( Fig. 1 D). Hence, we considered that the lesion may involve the pulmonary vein and extend to the LA. Endobronchial ultrasound (EBUS) uncovered an irregular occupying lesion in the basal segment of the lower lobe of the left lung. Brush examination demonstrated numerous neutrophils without malignant tumor cells. However, due to obvious puncture bleeding, no biopsy was performed.

**Fig. 3:**
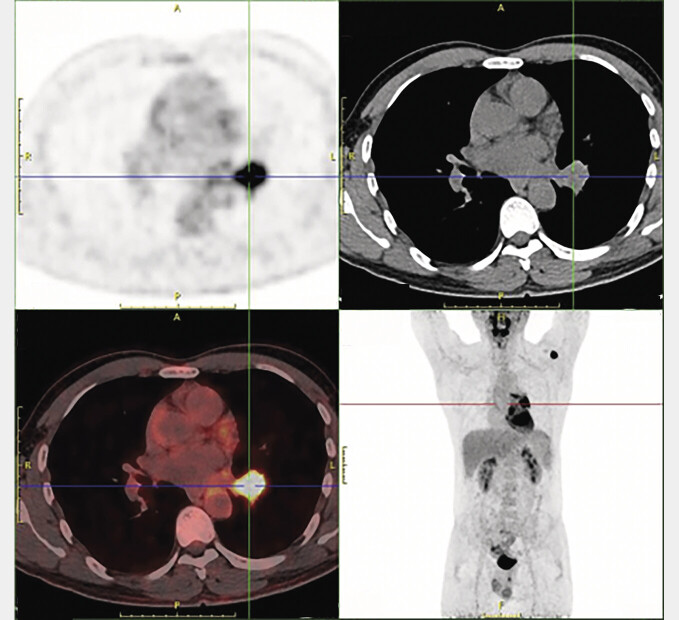
PET-CT showed hypermetabolic lesions in the lower lobe of the left lung adjacent to the hilum.

**Fig. 4:**
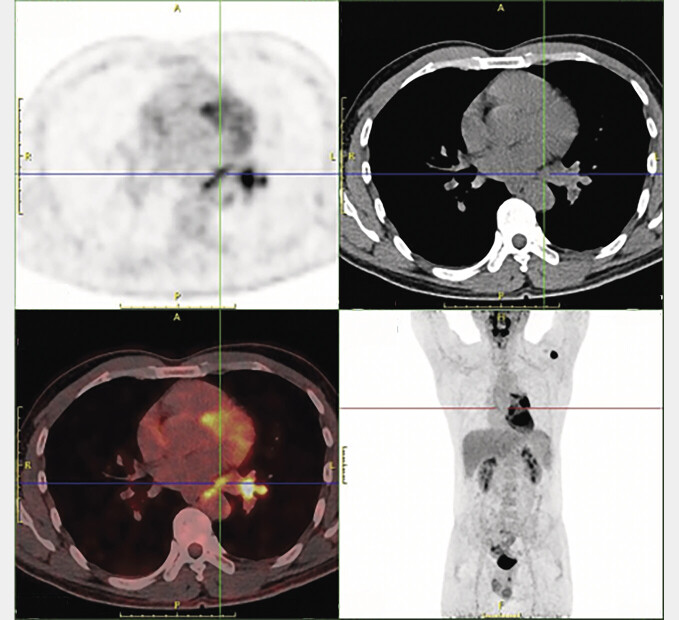
PET-CT detected a hypermetabolic lesion in the left inferior pulmonary vein.

Based on these findings, our attention was focused on the nature of the lesion: inflammation or tumor? Therefore, anti-inflammatory therapy was administered, but his symptoms showed no significant improvement. Hence, we considered the lesion to be a tumor. F18-fluorodeoxy glucose-positron emission tomography (18F-FDG PET CT) was performed. It showed hypermetabolic activity in the soft-tissue nodule in the left lung near the hilum with an SUVmax of 9.8 ( Fig. 3 , Fig. 4 ), in the left hilar lymph node with an SUVmax of 6.0, and in the left humeral head with an SUVmax 15.7. Based on the results of PET-CT, the possibility of a malignant tumor was considered and surgery was performed. The patient underwent left upper lobe lingular segmentectomy, lower left sleeve lobectomy, and subcarinal lymph node dissection. The mass extended from the left inferior pulmonary vein (LIPV) to the left atrium (LA). Intraoperative frozen pathology revealed interstitial fibrous tissue hyperplasia with lymphocytes, plasmocytes, and giant cells infiltrated by the lesion. Immunohistochemical results showed characteristic features of S-100(+), CD68/ PGM1(+), CD1a(–). Therefore, the lesion was identified as the Rosai-Dorfman disease. The irritating cough improved after discharge, and the patient became better and was followed up in the outpatient clinic.

Rosai-Dorfman disease (RDD) is a sinus histiocytosis named by Rosai and Dorfman. The etiology of RDD is unknown. Its prevalence is 1:200,000 and the mean age of patients is 20.6 years. The typical symptom is bilateral painless cervical lymphadenectasis and may involve mediastinal, groin, and retroperitoneal lymph nodes. About 43% of patients present with extranodal invasion [Elbaz Younes I et al. Cancers (Basel) 2022, 14(21)], such as skin, liver, lung, mediastinum, bone, etc., so patients have diverse clinical presentations [Yu L et al. Asian J Surg 2021, 44(11) **:** 1416–1417] [Oramas DM et al. Pathol Res Pract 2022, 234 **:** 153917] [Ren H et al. Front Oncol 2024, 14 **:** 1408353]. The diagnosis of RDD requires a combination of clinical manifestations, imaging, and pathology testing. Pathological diagnosis is based on histiocytic features, such as S100+, CD68+, and CD1a–. The clinical outcome of RDD has ranged from self-limited disease to disseminated refractory illness and death [Elbaz Younes I et al. Cancers (Basel) 2022, 14(21)].

In this case, hypermetabolic lesions were demonstrated in the lung, para-descending aorta, pericardium, and pulmonary veins on PET-CT. Finally, RDD was confirmed by pathology. The mystery had been solved even though the PET-CT results showed that the lesion was a malignant tumor with metastasis. In fact, RDD is a benign tumor with aggressive growth, possessing malignant characteristics. Bone involvement has been diagnosed in less than 10% of these patients. However, bone involvement in RDD has been underestimated due to its subclinical presentation, and PET-CT is more sensitive for detecting it [Chen LYC et al. N Engl J Med 2024, 391(12) **:** 1140–1151]. Hence, we speculated that the abnormal bone density of the left humerus may be caused by RDD.

Thus, we can learn more from this case. Firstly, multisection ultrasound scanning should be performed to confirm whether there was an occupying lesion or not. Secondly, we can use CDFI to evaluate flow characteristics and pulsed-wave Doppler to assess the maximal blood flow velocity. Thirdly, transesophageal echocardiography (TEE) examination is superior to TTE and can provide clear imaging because of its multiple sectional views. Last but not least, it should be noted that the gold standard for diagnosis is pathological analysis which can be conducted by the biopsy.

In conclusion, RDD is a rare and benign tumor, and the diagnosis requires multidisciplinary cooperation. Multiple imaging techniques play an important role in the diagnosis of RDD. Puncture biopsy can provide the final accurate diagnosis in this disease.

